# 3D Active Stabilization System with Sub-Micrometer Resolution

**DOI:** 10.1371/journal.pone.0042733

**Published:** 2012-08-10

**Authors:** Olli Kursu, Tuomas Tuukkanen, Timo Rahkonen, Mikko Vähäsöyrinki

**Affiliations:** 1 Department of Electrical and Information Engineering and Infotech Oulu, Electronics Laboratory, University of Oulu, Oulu, Finland; 2 Department of Physics, University of Oulu, Oulu, Finland; Claremont Colleges, United States Of America

## Abstract

Stable positioning between a measurement probe and its target from sub- to few micrometer scales has become a prerequisite in precision metrology and in cellular level measurements from biological tissues. Here we present a 3D stabilization system based on an optoelectronic displacement sensor and custom piezo-actuators driven by a feedback control loop that constantly aims to zero the relative movement between the sensor and the target. We used simulations and prototyping to characterize the developed system. Our results show that 95 % attenuation of movement artifacts is achieved at 1 Hz with stabilization performance declining to ca. 70 % attenuation at 10 Hz. Stabilization bandwidth is limited by mechanical resonances within the displacement sensor that occur at relatively low frequencies, and are attributable to the sensor's high force sensitivity. We successfully used brain derived micromotion trajectories as a demonstration of complex movement stabilization. The micromotion was reduced to a level of ∼1 µm with nearly 100 fold attenuation at the lower frequencies that are typically associated with physiological processes. These results, and possible improvements of the system, are discussed with a focus on possible ways to increase the sensor's force sensitivity without compromising overall system bandwidth.

## Introduction

Environmental or user-generated vibrations can be detrimental in measurements that require stable contact at the (sub)micrometer scale between the measurement probe(s) and its target. Such applications are becoming increasingly common in metrology, microelectronics and cellular level measurements from biological tissues. Stability at the scale of few micrometers is often difficult to achieve by environmental vibration isolation, especially if user has to handle the instruments during the measurements. Handling may create complex and multi-dimensional movement artifacts that affect the measurement probe and/or the target. Additional challenges can be faced in biological applications with living animals, such as recordings of the electrical brain activity, where additional target movements are generated by normal physiological processes.

Even when an experimental animal is securely fixed to the experimental setup to prevent its movements the brain undergoes constant micromotion that makes recording electrical activity of the nerve cells challenging. This micromotion results from periodic physiological processes, such as cardiac and respiratory functions, and transient movements generated by the activity of muscles in the head. It varies from few micrometers in small animals (e.g. flies [1]) to few tens or hundreds of micrometers in larger animals (e.g. rats [2] and cats [3]). In general, few micrometers of tissue movement will prevent stable single cell recordings from small cells and more than 5 µm typically leads to loss of recording also with larger cells [1], [2]. Instead of common method of eliminating the movement sources by extensive surgical procedures, an active stabilization system can be implemented to reduce the relative movement between the measurement electrode and the tissue. Successful demonstration of active stabilization based on the physiological signals [2], [4] or direct measurements of the brain micromotion have been previously presented [2]. However, these methods are constrained to one dimensional movement along the electrode axis, which may limit their general use.

We have developed an active 3D stabilization system to actively compensate for the movement artifacts. The system that we developed is described in detail and characterized with measurements and simulations. We also demonstrate active stabilization of complex movement trajectories derived from experimental measurements of blowfly brain micromotion [1]. Finally, we identify the major advantages and limitations of the system, and discuss possible future improvements.

## Materials and Methods

### System and mechanics design

The design concept of the 3D active stabilization system is based on a touch-probe type displacement sensor and a proportional-integral (PI) control loop that constantly aims to zero the measured movement by driving custom piezo-actuators. Inspired by the results of earlier work [1], the displacement sensor was designed to be based on photo interrupters (EE-SX1107, Omron Corporation). The photo interrupter is a fork-shaped component that has a light source (constant current light-emitting diode) and a photo detector that detects the movement of a light blocking element between the prongs of the fork.

**Figure 1:**
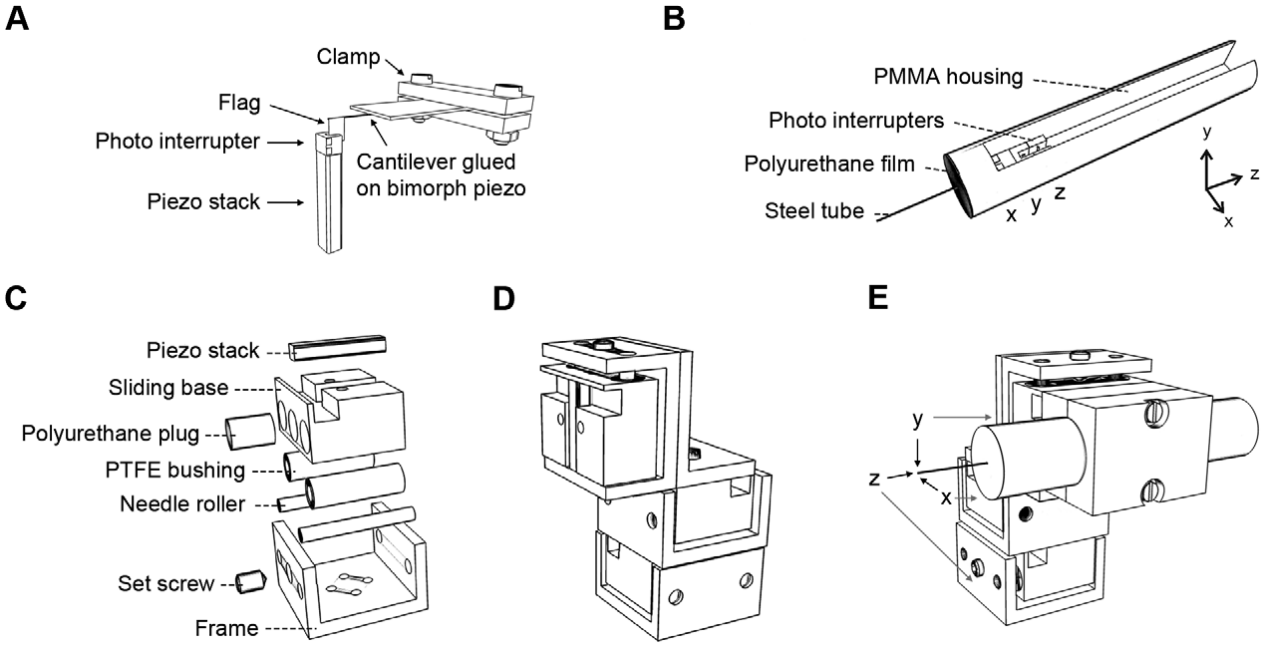
Schematic overview of the active stabilization system. A, 1D stabilization system prototype. B, 3D movement sensor prototype (diameter 12 mm and length 55 mm); axis definitions as used throughout the study. C, Piezo-actuator prototype. D, Three axis actuator assembly (25x37x56 mm^3^). E, Schematics of the stabilization system test setup with three-axis actuator and 3D movement sensor.

A simple 1D stabilization system prototype was first assembled to characterize control loop performance in isolation from complex mechanics of the 3D system. This was accomplished by gluing one photo interrupter directly on top of one stacked piezo component (PSt 150/2x3/20, Piezomechanik) that provides up to 28 µm movement in semi-bipolar excitation (-30 V to 150 V) and a high resonance frequency of 50 kHz (Figure 1A). A light blocking element, an aluminum foil flag, was glued on a short cantilever that was moved by a piezoelectric bimorph actuator (BIMP30/12/0.6-PX5-N, Morgan Electro Ceramics) in between the two prongs of the photo interrupter (Figure 1A). The bimorph actuator was controlled by a function generator (33220A, Agilent Technologies).

The 3D displacement sensor assembly consists of three photo interrupter components, which are glued back-to-back and a hole is drilled through the blocks to accommodate the probe (Figure 1B). The probe pierces a flexible polyurethane film that acts as a spring-like element. The 3D sensor was assembled to a housing machined from Poly(methyl methacrylate) (PMMA). The flexible film with 10 mm diameter was molded from a polyurethane elastomer (UR2440, Axson Technologies) and attached to the outer rim of the housing. Steel tubing with outer diameter of 310 µm was used as the probe (130 µm inner diameter) and an aluminum foil flag was glued to its back-end to enable covering the slit of the z-axis photo-interrupter. The photo detector slits were 150 µm in width and 0.6 mm in height, which made the steel tube probe itself large enough to act as a light blocking obstacle for the x- and y-axis (Figure 1B).

The 3D actuator for the stabilization system was built around three stacked piezo components (same as used in the 1D prototype). Each piezo stack was attached to a sliding base, which was attached to the frame with M3 needle rollers and tight fit PTFE bushings (Figure 1C). The actuator frame and the sliding base were machined from 7075 aluminum alloy. The sliding base moves along the PTFE bushings when pushed by the piezo stack. Although the stacked piezo components offer very high force production for pushing, they require pre-loading with the springs for a reliable return. This was implemented by the polyurethane elastomer molded plugs and a M4 conical set screw that enabled adjusting the pre-load (Figure 1C). The actuators were assembled into a three-axis configuration to enable 3D stabilization (Figure 1D). The 3D sensor was mounted on the y-axis of the three-axis actuator using a clamping piece (Figure 1E).

Characterization and testing of the 3D stabilization system was performed on a vibration isolated table (model no. 63-534, Technical Manufacturing Corporation). Manual xyz-positioning stages were used to pre-align the sensor and actuators to within their operating ranges (M-462, Newport Corporation). For the force sensitivity measurements, a calibrated force sensor (LTS-50GA, Kyowa Electronic Instruments) was attached to a commercial closed-loop nano-positioning system with 2 nm resolution (Tritor 100 SG with ENV 40 SG amplifier, Piezosystem Jena) and pushed against the probe tip. The resulting signals were measured with a multimeter (model no. 2000, Keithley Instruments). The movement trajectories of the nanopositioning system were produced either by a function generator or by a PC-based data acquisition system (USB-6211 with 6218 BNC, National Instruments). The control signals were amplified with a custom voltage amplifier to a suitable voltage range. Actuator displacements were measured with a calibrated commercial displacement sensor (optoNCDT 2401, Micro-epsilon). For the stabilization performance tests, the probe tip was rigidly glued to the commercial nanopositioning system.

### Electronics

**Figure 2:**
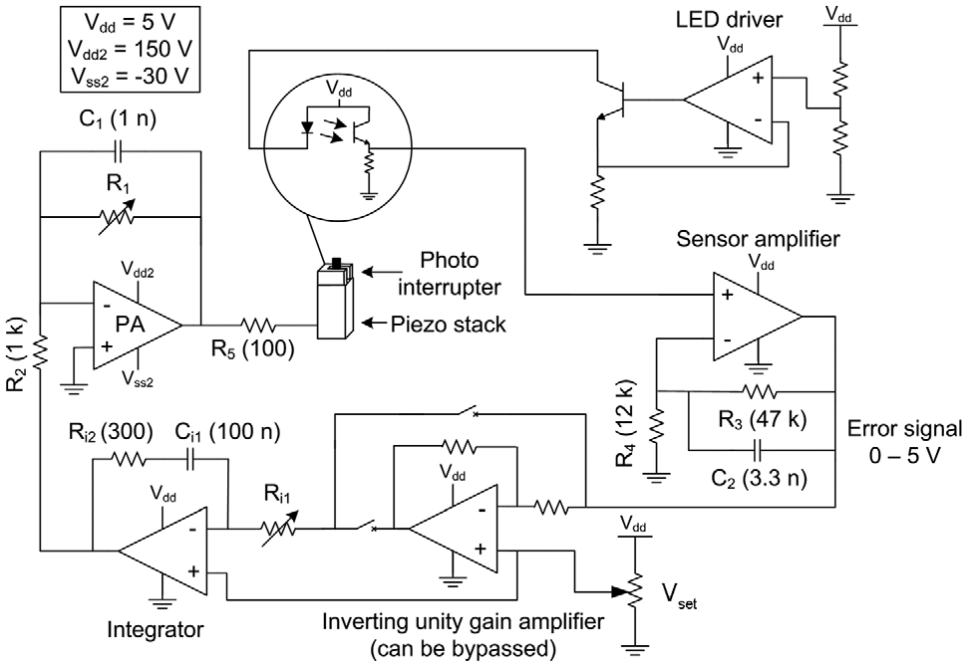
Schematics of one axis of the stabilization system electronics. PA  =  power amplifier; V_set_ is a parameter used to match the control loop DC level with that of the sensor output.

In the developed 3D displacement sensor (Figure 1B), the light is partially blocked either by the probe shaft or by the flag. The probe is aligned so that the linear region of the photo interrupter output is used (between 20 % and 80 % of the operating range). This corresponds to an output signal of 0.2 – 1 V, which is further amplified to 1 – 5 V range (Figure 2).

Design of the developed PI control loop (Figure 2) enables tuning the integrator time constant and gain with trimmers (power amplifier: PA240CCs, Cirrus Logic; low voltage amplifier: AD8629s, Analog Devices). Dc level at the power amplifier output can also be adjusted with a trimmer (not shown) connected to the positive input. 150 V supply voltage for the power amplifier is provided by switching dc-dc converter (1/4A12-P4, Ultravolt) and -30 V negative supply by switching inverting dc-dc converter (LT3462, Linear Technology). Depending on the sensor pre-alignment (i.e. on which side of the photointerrupter slit the light blocking obstacle is pre-aligned to) the same movement can produce increasing or decreasing signals. This could in effect transform a negative feedback loop into a positive one and, therefore, an inverting unity gain amplifier that can be bypassed with a jumper was also added (Figure 2).

### Modeling

**Figure 3:**
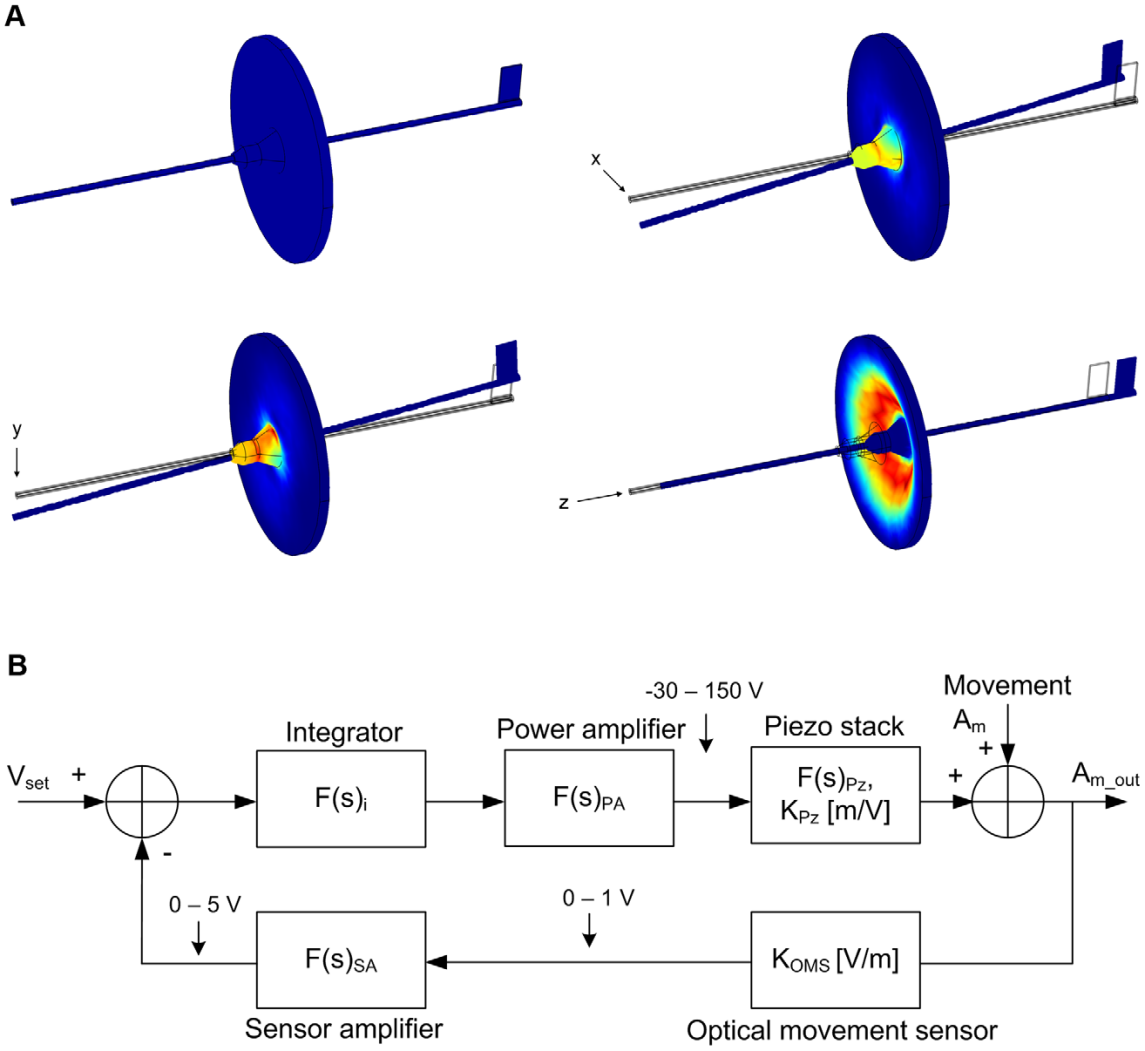
Modeling and simulations. A, Simulations of the 3D sensor movement in response to manipulation of the probe tip; colors illustrate strain of the film. B, Schematic representation of the linear model of one axis of the stabilization system control loop.

A mechanical model of the 3D sensor prototype was developed using a finite element method (FEM) [5] and implemented with the COMSOL Multiphysics® software (COMSOL AB). Because the main purpose of the modeling was to study the mechanical properties of the flexible film and the probe, the sensor housing and photo interrupters were excluded from the simulations (Figure 3A). Otherwise the mechanics was modeled using the dimensions and material properties of the prototype (Table 1). The polyurethane film thickness in the simulations was adjusted to 0.5 mm to match the measured force sensitivities of the prototype. A Neo-Hookean material model was used in the simulations for the polyurethane film and an isotropic material model for the steel tubing.

**Table 1:** Parameters used in the FEM model.

Parameter	Film	Probe shaft
Material	polyurethane	stainless steel
Diameter (mm)	10	0.31 (outer), 0.13 (inner)
Thickness (mm)	0.5	
Length (mm)		24
Young's modulus (GPa)	0.006	193
Poisson's ratio	0.49	0.3
Shear modulus (MPa)	2.2	
Bulk modulus (MPa)	100	
Density (g/cm^3^)	1.02	8

The stabilization performance of the system was also linearly modeled (Figure 3B). For the 1D prototype (Figure 1A), gain of the sensor, K_OMS_, was measured to be approximately 3.10 mV/ µm. The piezo stack was modeled with a 447 nF capacitance based on the results from the magnitude and phase measurements with an impedance analyzer (LCR HiTester 3522-50, Hioki E.E. Corporation). Gain of the piezo stack constant, K_Pz_, represents the elongation in response to the applied voltage, which according to the manufacturer's datasheet is 156 nm/V. The models were developed and implemented in MATLAB® (MathWorks) and simplified to only include the low frequency response and single pole transfer functions (Figure 3B). The open loop transfer function, F(s)_OL_, for the simplified system can be calculated according to:



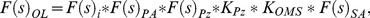
(1)where the PI controller transfer function, F(s)_i_, is
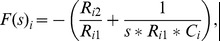
(2)the power amplifier transfer function, F(s)_PA_, is 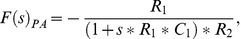
(3)the combined transfer function, F(s)_Pz_, of R_5_ and the piezo stack (Figures 2 and 3B) is
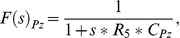
(4)and the sensor amplifier transfer function, F(s)_SA_, is 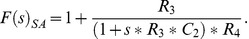
(5)


The closed loop transfer function, F(s)_CL_, for the compensated movement can be calculated as: 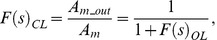
(6)where A_m_ is the movement signal and A_m_out_ is the residual movement signal after the stabilization (Figure 2). Finally the stabilization performance can be calculated in terms of attenuation percentage as:
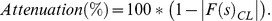
(7)


The 3D stabilization system was modeled for each of the axis separately using similar procedures as described above. It should be noted that assumptions of the model do not take into account the resonances within the systems, which limits its use to low frequencies.

## Results

The goal of this study was to develop an active stabilization system that is able to compensate for movement artifacts occurring during the measurements. The performance goal for the system was to attenuate movements that are below 20 µm in magnitude and slower than 10 Hz in dynamics to the 1 µm residual level. These specifications match the requirements of the selected example application of brain micromotion stabilization [1]-[4].

**Figure 4:**
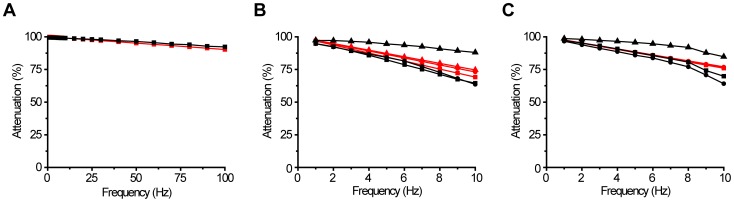
Simulated and measured stabilization performance. A, Measured (black) and simulated (red) movement attenuation performance of the 1D system. B, Measured (black) and simulated (red) movement attenuation performance of the 3D system for x (squares), y (circles) and z (triangles) with 5 µm movement C, Same as in B but for 10 µm movement.

A previously characterized 1D displacement sensor prototype [1], [6] was first assembled (Figure 1A) to test the developed PI control loop (Figure 2) together with the selected piezo component in isolation from the complex mechanics of the 3D system (see Materials and Methods for technical details). Stabilization performance was measured by actuating the light blocking obstacle between the photointerrupter prongs with a 10 µm peak-to-peak sinusoidal movement of varying frequency, while trying to zero this movement with piezo-component driven by the control electronics (see Materials and Methods). 99 % attenuation was achieved at the frequencies below 10 Hz after tuning the PI loop (R_i1_ = 205 Ωand R_1_ = 56 kΩ; Figure 2). The attenuation level declined slowly towards higher frequencies still remaining above 90 % at 100 Hz (Figure 4A). The high performance validates use of the selected components and successful implementation of the control loop.

### Displacement sensor and actuator characterization

A novel displacement sensor design was developed that was inspired by our earlier work [1]. It is based on an elastomer film that acts like a spring-like element and a steel tube that acts as a rigid probe (Figure 1B; see Materials and Methods). The flexible film allows a linear movement of the probe along the probe axis and a seesaw like movement perpendicular to it (Figure 3A). Ramp displacement signals with maximum amplitude of 10 µm were used to calibrate the displacement-voltage relationship. The 3D sensor was found to be linear within the measurement range with displacement sensitivities of 14.1 mV/ µm, 18.0 mV/ µm and 11.8 mV/ µm for x, y and z-axis, respectively. This corresponds to approximately 100 nm movement resolution, assuming that signals larger than 1 mV can be reliably measured in laboratory conditions [1]. In addition, force sensitivity of the sensor was determined and found to be 3.59 µN/ µm, 3.77 µN/ µm and 211 µN/ µm for x, y and z-axis, respectively. The force sensitivities were also linear within the tested 80 µm movement range.

Two general observations were made during the sensor characterization that deserved closer inspection. First, some cross-axis influences were observed in the movement sensing that ranged from 4 % to 25 %, depending on the pairs of axis (Table 2). Second, there was a large difference in the force sensitivities between the x- or y-axis and the z-axis. A FEM model was developed for the sensor mechanics to study the cause of these findings (Figure 3A; see Materials and Methods). Simulations were made where tip of the sensor probe was displaced along one of the three axes. Results of the simulations showed that the probe shaft moved only along the axis of the induced movement (Figure 3A), ruling out the possibility that the observed correlations are inherent to the basic design of the sensor. Instead, a closer examination suggested they are likely to be result of inaccuracies in the prototype assembly and/or from inaccurate alignment of the sensor's axis to those of the commercial nanopositioning system used in the calibration. Although the cross-axis influences will affect the absolute accuracy of the measured movements, absolute information is not required for the compensation system to work properly (i.e. low amount of axis-to-axis coupling will not jeopardize the control loop stability). Analysis of the simulation results also showed that the varying force sensitivity between different axes was not attributable to bending or buckling of the probe shaft under forces caused by the tip displacements. Instead, it resulted from the elastic properties of the film that poses a much higher spring constant for the probe movement along the sensor's principal axis than perpendicular to it (Figure 3A). The limited force sensitivity of the z-axis and possible means to improve it are further addressed in the Discussion.

**Table 2:** Cross axis correlations (displacement amplitude is given on top of each column).

x = 10 µm	y = 10 µm	z = 10 µm
x = 2.87 mV/ µm	x = 0.16 mV/ µm	x = 0.24 mV/ µm
y = 0.45 mV/ µm	y = 3.66 mV/ µm	y = 0.10 mV/ µm
z = 0.16 mV/ µm	z = 0.93 mV/ µm	z = 2.40 mV/ µm

**Figure 5:**
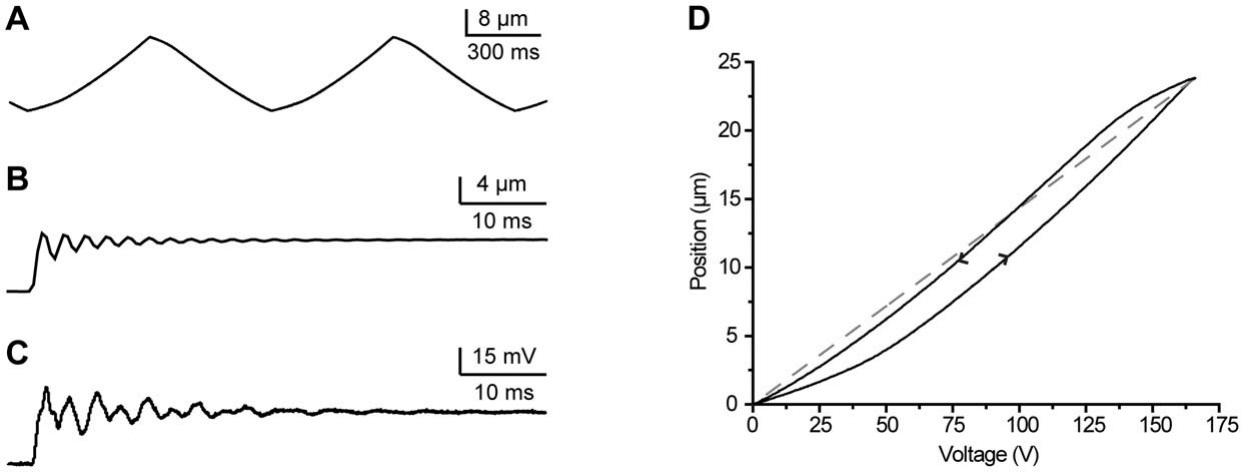
Dynamical system characterization. A, Actuator movement response to a triangle wave control signal. B, Actuator displacement in the z-axis in response to a square wave control pulse. C, Sensor resonance in response to a square wave probe tip displacement. D, Actuator hysteresis determined from A; dashed line represents an ideal case with no hysteresis.

Next, dynamic properties of the custom-made piezo-actuators (Figure 1C, 1D; see Materials and Methods) and the 3D displacement sensor were characterized. Triangle wave signals were first used to drive the actuators to quantify the hysteresis (Figure 5A), a deviation in the position between the forward and backward movements, known to affect the stacked-piezo component based actuators. The resulting input voltage – position curve showed only a moderate hysteresis (Figure 5D), which is unlikely to affect compensation system performance because of the high control loop gain. Next, piezo-actuators were driven with step-like fast displacements and the resulting movements were measured with a calibrated commercial displacement sensor. Damping oscillations with a single frequency were observed that varied slightly for each actuator from 320 to 400 Hz (Figure 5B). Because resonance frequencies of the piezo-components themselves were much higher, the observed resonances are attributable to the spring constant of the pre-loading spring and masses within the actuator assembly. It should be noted that slight variation in the resonance frequency is to be expected attributable to the individual hand-tuning of the pre-load for each actuator. To complete the dynamical characterization of the system, resonances within the sensor were elicited by displacing the tip of the sensor probe rapidly in a step-like fashion with the commercial nanopositioning system. Clear oscillations were observed that consisted of three main frequencies around 180, 330 and 1200 Hz (Figure 5C). Simulations with the sensor's FEM model were used to identify the cause for these resonances. Results showed that 180 Hz and 330 Hz correspond to the resonance of the polyurethane film for movements in x/y-axes and z-axis, respectively, and that 1200 Hz was attributable to the probe shaft resonance.

### Stabilization performance

**Figure 6:**
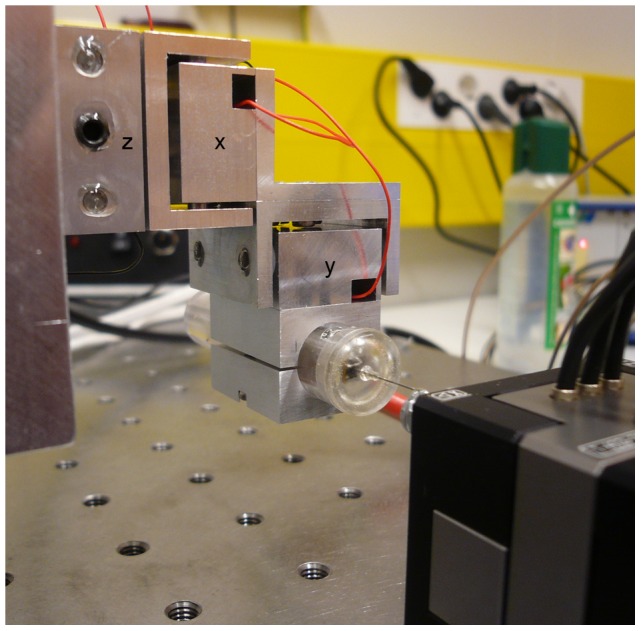
Photograph of the 3D stabilization system prototype. The three-axis actuator is mounted sideways to a vertical platform and the 3D movement sensor is attached to the last actuator of the assembly; the probe tip is glued to the commercial nanopositioning system at the bottom right corner Axis definitions are placed to each of the actuator axis to enable comparison to schematic presentation in Figure 1E.

Stabilization performance of the 3D system was measured by moving tip of the sensor with the commercial nanopositioner, while trying to zero this movement with the developed system (Figures 1E, 6). Sinusoidal movements with varying frequencies were simultaneously generated for all three axes. After tuning the PI control loop (R_i1_ = 1.8 kΩ and R_1_ = 35 kΩ; Figure 2), attenuation performance exceeded 95 % at 1 Hz for all three axes with both 5 and 10 µm movement signals (Figure 4B, 4C). The performance declined with faster movements to ca. 85 % for the z-axis and to ca. 65 % for the x- and y-axes at 10 Hz (Figure 4B, 4C).

A linear model of the compensation system was developed (Figure 3B; see Materials and Methods) to study the cause for the observed drop in the stabilization performance with higher frequencies when compared to the simple 1D system. Simulations with model of the 1D prototype showed a close match with the experimental results validating the modeling approach used (Figure 4A). Because the control loop implementation as well as the photointerruptors and piezo-components were the same for the 1D and 3D systems, the decreased stabilization performance was expected to result from the lowest resonance frequencies of the sensor's elastic film, as characterized earlier. Indeed, when tuning the control loop, oscillations indicating instability of the 3D stabilization system were observed with the lower bandwidth and gain settings than with the 1D system, which were attributable to the sensor resonance. This will necessarily lead to reduced stabilization performance, which was also confirmed with the simulations of the 3D system (Figure 4B, 4C). Although the linear model of the more complex 3D system was not able to reproduce the experimentally determined performance as well as with the simple 1D system, the simulations show consistent decreasing trend in the stabilization performance with the higher frequencies.

**Figure 7:**
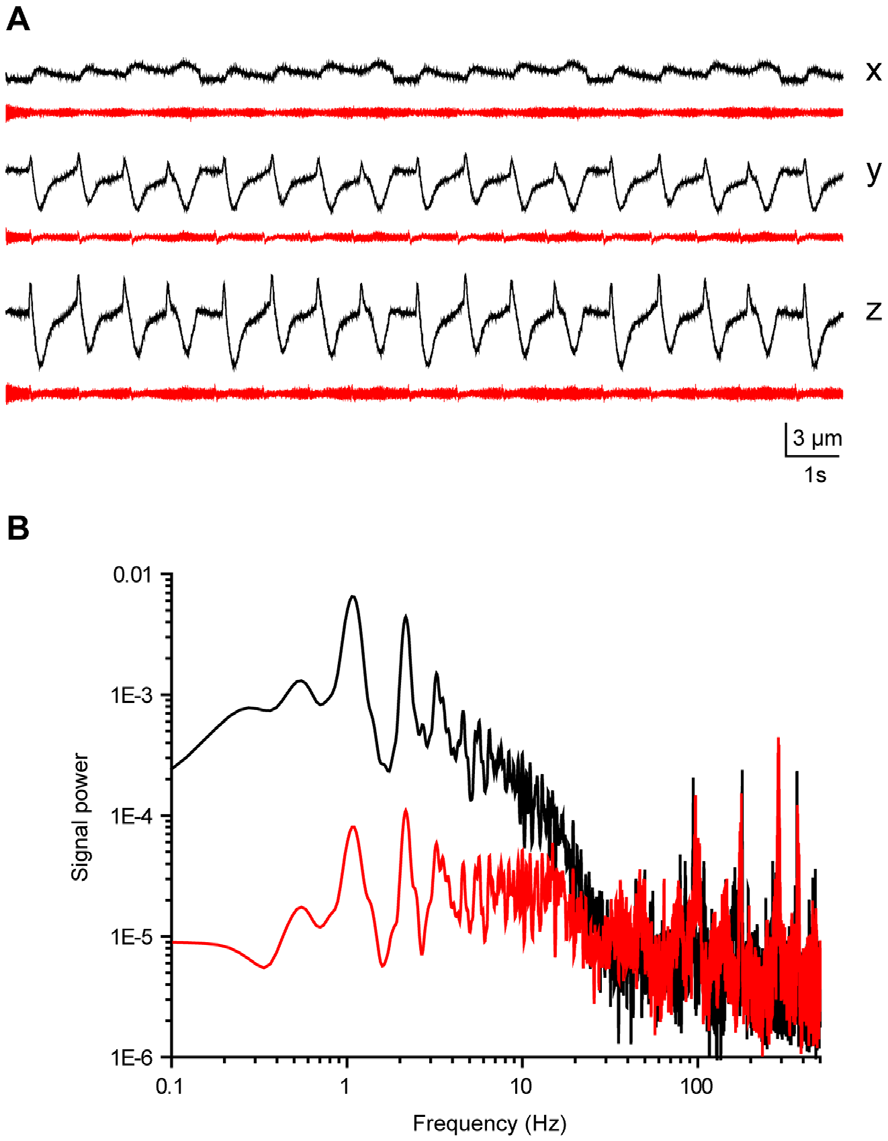
3D stabilization of the brain derived micromotion sequence. A, Movement relative to the sensor tip before (black) and after the stabilization compensation (red) for each of the axis. B, Power spectrum of the z-axis data in A.

Finally, brain derived micromotion was used to demonstrate the stabilization system performance with complex movements. The traditional approach to circumvent the brain micromotion related problems in experiments has been to anesthetize the animal and/or eliminate the movement sources through extensive surgical procedures. However, such invasive procedures may affect function of the cells under study (see e.g. [7]-[9] for recent accounts on the topic). The active stabilization approach could provide a less invasive solution for the prevailing problem, making it a topical example application. Although the 3D movement sensor design had advantages over our previous designs [1] the compromised force sensitivity prevented its use with the small animal models and soft tissues (< few hundreds of nN/ µm would be recommended [1], [10], [11]). Therefore, previously published blowfly (*C. vicina*) brain micromotion data [1] was used to move the sensor tip with the nanopositioning system, while the sensor was attached to the 3D actuator assembly (Figures 1E, 6). Although the rigid mounting of the sensor tip differs from the compliant coupling that would result from insertion of the tip to the biological tissue the test arrangement provided a technically well controlled setting for characterizing the stabilization performance (see also Discussion). A typical micromotion sequence of 3.7 s in duration was selected from the published data that shows rhythmic brain movements attributable to the respiration (Figure 7A). The sample sequence was also selected in a way that the dc-level did not significantly change from the beginning to the end, which enabled continuous looping of the sequence. Ca. 5 µm micromotion was successfully stabilized down to 1 µm level (attenuation of 36%, 75% and 77%, for the x-, y- and z-axis, respectively), which meets the set performance target. It should be noted that the resulting attenuation percentages are largely limited by the signal-to-noise ratio of the sensor output. Similar stabilization level with 1 µm level residual would be expected also for the larger movements, especially for those resulting from relatively slow physiological processes (Figure 7B; [1]-[4]).

## Discussion

We have developed and demonstrated an active stabilization system for the movement artifacts. The performance goal were set in accordance with the requirements for stabilizing brain micromotion in small animals to enable stable single cell recordings [1], [2]. The goal was achieved both in the charazterization tests with simple motion trajectories as well as with the experimentally derived [1] brain micromotion trajectories [1]. As described in the results, the insufficient force sensitivity of the sensor limited brain micromotion stabilization tests to somewhat artificial configuration: the sensor tip was rigidly attached to the commercial nanopositioner with high stiffness. This differs from the situation in the brain tissue where small volume of soft and slightly elastic tissue would move together with the tip. Since exact character of the tissue coupling will be different in each experiment depending on the animal model and preparation, as well as on the exact measurement geometry and damage attributable to the insertion, we decided not to attempt to model the coupling. The additional mass coupled to the tip would lower the resonance frequencies, but at the same time the tissue is lossy by its character, effectively damping the possible resonances (unpublished observations of earlier work [1]). This means that more gain could be used in the control loop and, therefore, similar level of attenuation may be achievable despite of the compliant coupling in the brain tissue.

Few examples of successful demonstrations in the brain micromotion stabilization have been published previously [2], [4], but the earlier systems were limited to linear motion along the electrode axis. The 3D stabilization may be important for the experiments where motion perpendicular to the measurement electrode axis would prevent stable cellular recordings (e.g. *in vivo* patch-clamp recordings). In addition, the 3D information of the ongoing movements may be beneficial to compensate for in the biological imaging applications, where signal is based on the repetitive scanning in 3D [12]. The developed system could be implemented in the experimental setups by integrating both the displacement sensor and the experiment specific measurement probe(s) into one assembly attached to the piezo-actuators. As an alternative, the measurement target could be placed on the piezo-actuators. The displacement sensor and the experiment specific measurement probe(s) could even be installed separately if the relative coordinates between the target and those of the sensor and probe(s) are known, which would enable implementing the coordinate transform to the control loop. More detailed analysis of the advantages and disadvantages in comparison to the previous work is topic of the future work when our system is developed further and validated with the animal experiments.

An analog PI control loop was selected for the compensation, which is simpler to implement and typically faster with a wider bandwidth than a digital one. These benefits were judged to be more important than the related disadvantage of the limited flexibility for the tuning and re-configurability. A PID-type control loop could potentially provide better performance, but it would be more difficult to tune and the derivative term could also introduce gain at high frequencies, thereby amplifying the high frequency resonances and noise. The electronic implementation of the control system could be further improved by a more sophisticated designs aimed to suppress the feedback gain at the frequencies of the mechanical resonances within the system (note that such lead-lag type features are already implemented in the control loop to some extent by the transfer function zero of the sensor). However, our results show that the control system electronics was already outperforming the mechanical implementation of the system that created the real bottle-neck for the stabilization performance (e.g. Figure 4A vs. 4B). On the other hand, detection resolution of the system was limited by the noise within the optoelectronic sensor output, thereby, setting a limit for the achievable stabilization resolution. Despite of the noise, it was still possible to achieve 1 µm stabilization level in typical office settings for ambient noise.

**Figure 8:**
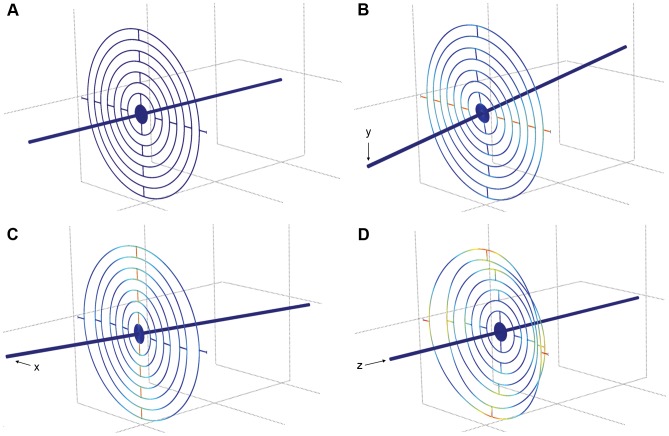
FEM simulations of the concentric arc sensor design. A, Simulated design in rest. B, Simulation of the probe tip movement along y-axis. C, Simulation of the probe tip movement along x-axis. D, Simulation of the probe tip movement along z-axis. Colors illustrate the strain of the metal film.

Although the pre-loading spring and the mass of the actuators created mechanical resonances, the system performance was limited by the lower resonance frequencies within the sensor. This is also illustrated by the significantly higher compensation performance on the z-axis, where sensor's resonance frequency is roughly twice of that of the x- and y-axes. Optimization of the stabilization bandwidth vs. high force sensitivity of the presented sensor design was found to be especially challenging. Higher force sensitivity would have been required especially at the z-axis for the brain micromotion compensation in the small animal [1], but this would have lead to lower resonance frequencies and compromized stabilization performance. As a starting point for future work, a thin sheet metal based film designs were simulated that have better weight-to-stiffness ratio than the urethane film. Steel was used in the simulations as a material and different geometries were tested to find a design where sensitivity of the z-axis can be adjusted without significantly affecting the properties of the x- and y-axis. A concentric arc design was found to provide flexible tuning possibilities for the force sensitivity and the resonance frequencies of each axis by optimizing for the material thickness, line width and arc spacing ( = length of the straight parts between arcs; Figure 8). The simulations were made for a design with a 170 µm diameter quartz glass probe and 25 µm thick steel film with 50 µm line width and 500 µm arc spacing. The resulting force sensitivities were 45 and 720 nN/ µm for the x/y and z-axis, respectively, which meet the requirements set by measurements in the soft biological tissue [1], [10], [11]. Importantly, the several orders of magnitude improvement in the sensitivity in comparison to the urethane film based design was accompanied by only two to three-fold decrease in the resonance frequencies (x and y: 82 Hz; z: 120 Hz). Simulations also showed that this design was free from the cross-axis influences.

The concentric arc sensor design provides a feasible concept for future efforts aimed towards improving the sensor's force sensitivity without compromising the bandwidth of the system. Differences in force sensitivities between the x/y- and z-axis could also potentially be eliminated with a two element design [13]. It should be mentioned that a MEMS-based 3D force sensor with nN scale force sensitivity was recently described [14]. The presented compensation system would also work with such force sensor, where the control loop would aim to zero the movement artifact generated forces. However, multi-axis MEMS force sensors are currently hard to get, limited in their operating range and delicate, which limits their practical implementation possibilities. We believe that our approach provides, in general, a good platform for future developments of 3D active stabilization systems for technical and biological measurements.
